# Impact of Implementing New ICF-Based Practices on Staff Valence of Disability Practitioners: An Experience in Hong Kong

**DOI:** 10.3390/ijerph20021632

**Published:** 2023-01-16

**Authors:** Phyllis King Shui Wong, Cheuk Lun Kwan, Yu Cheung Wong

**Affiliations:** 1 Department of Social Work, The Chinese University of Hong Kong, Hong Kong, China; 2 School of Social Sciences, Caritas Institute of Higher Education, Hong Kong, China

**Keywords:** disability practitioners, ICF-based practices, intellectual disability, organizational culture, staff valence

## Abstract

The International Classification of Functioning, Disability, and Health (ICF) was endorsed by The World Health Organization (WHO) in 2001. However, Hong Kong is at the beginning stage of implementing and testing ICF-based practices. This study examines any changes in the valences of disability practitioners in an organization under the newly introduced ICF-based practices. It was hypothesized that the involved staff members’ self-perceived valences in relation to the ICF would be enhanced. A pretest-posttest design was adopted. The 27-item Scale on Staff Valence under ICF-based practice (SSV-ICF) was used to measure the impact on staff valence of a pilot scheme in which ICF-based practice was implemented. Self-report questionnaires were completed by the involved staff members at the beginning of the pilot scheme and 12 months later. Analyses used paired samples *t*-tests and one-way repeated measures ANOVAs, performed by SPSS software, version 25. In total, 91 participants took part in the study. Results showed that participants achieved positive changes in all domains of valences, while participants’ level of involvement in the new ICF-based intervention had significant effects on their score differences in the “Competence” domain (r = 0.262, *p* < 0.05), “Intrapersonal” domain (r = 0.242, *p* < 0.05), and “Total Score” of SSV-ICF (r = 0.210, *p* < 0.05). The study demonstrated that disability practitioners who implemented ICF-based practices developed higher staff valences, which, in turn, benefited service users. Implementation of ICF-based practices also contributed to a more positive organizational culture.

## 1. Introduction

The International Classification of Functioning, Disability, and Health (ICF) was released by The World Health Organization (WHO) in 2001. It is regarded as a paradigm shift, changing practices from a merely medical model to an integrated bio-psycho-social model in which human functioning and disability are redefined as the dynamic interaction between a person’s health condition, personal and environmental factors [1,2]. 

In organizations that provide services for people with disabilities, the ICF can be applied in clinical practice to facilitate information-sharing and communication beyond the mere diagnosis of health conditions, thus broadening the focus to include how a health condition can impact the life of an individual. It can also be employed in surveys to collect data for statistics that will be used in resource planning and development, quality improvement, management and outcome evaluation [1,2]. 

In terms of outcomes, the ICF aims to achieve a better quality of services [3] since the resulting services are better able to address the individual needs of people with disabilities. The emphasis placed by the ICF on activities and participation contributes to the goal of independent community living for people with disabilities [4]. In view of the holistic approach taken by the ICF, the ultimate outcome of this intervention is the enhancement of the quality of life for people with disabilities [5].

Various assessment tools and service practices have been developed worldwide and evaluated since the publication of the ICF, for example [6,7,8,9,10,11,12,13]. However, research on the impact that such implementation has on staff members themselves has been little studied or documented. A study in the Netherlands explored how the introduction of an ICF-based rehabilitation tool in rheumatology has had an impact on staff satisfaction [14]. Another study in Switzerland collected feedback from team members after they implemented the ICF in their daily practices of neurorehabilitation [15]. A recent study in the Netherlands identified the barriers and enablers in the path of team members implementing an ICF-based tool in clinical otology and audiology practice [16]. To the best of our knowledge, no similar study about the impact of implementing ICF-based practices on staff has been carried out in the Asian context. 

In Hong Kong, there are only a few non-government organizations (NGOs) stating that they have adopted the ICF framework and implemented ICF in their service practices. In fact, putting the ICF framework into practice requires changing staff members’ mindsets, which, to many conventional organizations, is a significant barrier. Many organizational change theories have highlighted that aside from factors related to the content and context of the change itself (such as how to implement it, how to monitor the process, what infrastructure is required, whether enough training is provided, etc.), there are important human factors, which may revolve around new role relationships, including staff members’ personal attributes (e.g., work expectations, job satisfaction), their confidence in their ability to change, their perception of the benefits and values of the change, their peer relationships, etc. [17,18,19,20,21,22]. If these human factors are properly managed, they have been shown to facilitate more efficient and effective organizational changes. Staff members have been found to be extremely influential with their service users, especially in disability-services organizations [23]. If staff members experience a positive internal organizational climate towards the change, the needs of service users can also be better satisfied [24]. Therefore, when organizations are considering adopting the ICF framework, the more understanding they have of the impact of ICF-based practices on their staff members, the better implementation strategies they can develop.

Acknowledging the importance of creating a positive organizational culture in adopting the ICF-based practices, as a first attempt in the Asian context, the present study had two objectives. First, the study aimed to examine the change in staff self-perceived valences under ICF-based practices. Staff self-perceived valences that the study sought to evaluate included: (1) Staff members’ self-assessment of their knowledge and competence in applying the ICF, (2) how staff members perceive the changes that will affect them as part of a multidisciplinary team, and (3) the perceived meanings about the use of the ICF-based practices [25]. It was hypothesized that, following the organization’s introduction and testing of ICF-based practices in its services, the involved staff members’ self-perceived valences in relation to the ICF would be enhanced. Second, the study aimed to explore if there are any personal factors, such as work experience, position, etc., that have a significant effect on those changes.

## 2. Materials and Methods

### 2.1. Study Site

A non-governmental organization (NGO) that provides a variety of services for adults with intellectual disabilities (ID) in Hong Kong was invited to participate in the study. This NGO was selected for the study for the following reasons: (1) The organization is one of three in Hong Kong stating that it is trying out the ICF model, and (2) the organization was already planning to implement a one-year pilot scheme to try out newly developed ICF-based practices in its service delivery, which made for very good timing with our pretest and posttest study. 

To participate in the pilot scheme, 37 service users from 27 service units of the organization were selected to try out its newly developed ICF-based practices. A transdisciplinary team was formed for each selected service user and was responsible for the following components of practice: (1) Assessing the service user’s health and disability conditions, (2) developing the rehabilitation/support plan for the service user, and (3) implementing the plan and conducting its evaluation, all from the ICF perspective. The size and members of each team were different, depending on the situation and service setting of its responsible service user. In total, 129 staff of the organization were involved. These included social workers, paramedical practitioners (i.e., nurses, psychologists, physiotherapists, occupational therapists, speech therapists), and direct support workers (i.e., wardens, rehabilitation workers, and vocational instructors). Their involvement in the ICF-based practices varied: Some were involved in the assessment and/or planning only, some were involved in the third component to implement the annual rehabilitation/support plan, and some were involved in all three components. 

Covering all three components, the organization had developed a set of administrative forms that adopted the ICF framework in reference to the WHODAS 2.0 [26] to facilitate and guide the teams in working with the service users according to the principles of ICF. Particularly, the new forms required the team to place more emphasis on assessing and enhancing the service user’s “Activity”, “Participation”, and “Environment” factors, which are the core elements under the ICF framework. The teams were also required to complete the forms. All team members received a series of structured ICF training on the concepts and principles of ICF and how to use the newly developed forms in their work.

### 2.2. Study Design

The pretest-posttest design was employed in this study to investigate if there was any change in the self-perceived valences of the participating staff members who were involved in the previously mentioned transdisciplinary team to implement the newly developed ICF-based practices. Pretest data were collected at the beginning of the pilot scheme and posttest data were collected 12 months later. 

Ethical approval was sought prior to the study from the Survey and Behavioral Research Ethics Committee of the Chinese University of Hong Kong (Reference No. SBRE-18-583), the home institution of the principal investigator.

### 2.3. Instrument

The 27-item Scale on Staff Valence under ICF-based practices (SSV-ICF) [25] was used to measure the impact that introducing ICF-based practices under the organization’s pilot scheme had on participating staff members. The SSV-ICF is a self-report scale constructed to measure staff’s self-perceived valences in relation to ICF in five psychological domains: (1) The “Knowledge” domain (4 items) measures how the participant perceives his/her understanding of the ICF model, (2) the “Competence” domain (3 items) refers to his/her self-perceived competence in applying the ICF framework in services, (3) the “Intrapersonal” domain (6 items) relates to how the participant perceives the personal values of his/her work gained from ICF-based practices, (4) the “Relational” domain (8 items) relates to how the participant perceives the values gained from the collaborative relationship with team members and service users under ICF-based practices, and (5) the “Meaning” domain (6 items) measures how much the participant believes in and agrees with the concept and values of ICF. All items had the same score range (1 to 7). All 27 items of the SSV-ICF can be seen in the reference material listed in the 25th entry of the References section. These ICF-related valence domains were examined via Exploratory Factor Analysis (EFA) and Confirmatory Factor Analysis (CFA) [25] through split-half samples. The SSV-ICF scale has demonstrated acceptable psychometric properties (*χ*^2^/*df* = 1.836, CFI = 0.966, TLI = 0.961, RMSEA = 0.072) and very high internal consistency (Cronbach’s α = 0.981) [25]. 

### 2.4. Data Collection

At pretest, the questionnaires were sent to the center managers of the 27 service units on 3 June 2019. The center manager of each service unit was responsible for distributing questionnaires to participating staff members. Staff members were invited to return the completed and sealed questionnaires with signed consent forms to their center managers. After the data collection period, the center managers put all sealed questionnaires in the provided self-addressed envelopes and returned them to the principal investigator. At posttest, the questionnaires were sent out to the center managers again on 30 May 2020. The data collection procedure was the same as the pretest one.

### 2.5. Participants

In total, 91 participating staff members (20 males and 71 females) had completed the SSV-ICF questionnaires at both pretest and posttest. All the participants were aged 21 or above, approximately 40% of them were 41–50 years of age. Of these, 68.2% had more than 10 years of experience in providing services for people with intellectual disabilities. Approximately 26.4% of them were direct support staff, while 39.6% were social workers and 34.1% were paramedical practitioners. Of the participants, 65 were working in the residential service setting, and the others (28.6%) were working in community-based services. Their socio-demographic characteristics are summarized in Table 1. As described in the previous section, study participants had different levels of involvement in trying out the new ICF-based practices: 64.8% of them were involved in one of the three components, while 35.2% were involved in two or more components. 

### 2.6. Data Analysis

The purpose of the analyses was to find out whether, after attempting to apply for a year the newly developed ICF-based practices in service units, there was any change in the participating staff members’ self-reported various valence domains, as discussed in Wong et al. [25]. If there was a significant change, the study was also designed to discover whether there was any personal factor affecting that change. 

The various domains were first examined separately using paired *t*-tests to identify whether and what changes occurred in the scores of the participants, as measured at pretest and posttest. Then, one-way repeated-measures ANOVAs were used to explore any personal factors that might have significant effects on the changes, including gender (“male/female”), age (“21–30/31–40/41–50/51–60/60+”), position (“social workers/paramedical practitioners/direct support staff”), years of experience in services for people with intellectual disabilities (“10 or less/11–20/more than 20”), and service settings (“residential/non-residential”). We also assessed as two separate covariates the impacts of the level of involvement in the practices (from 1–3, i.e., 3 levels ranging from “involved in transdisciplinary team meeting”, “in support plan meeting”, and/or “in the process of assessment, planning, and implementation of the support plan”) and the number of the ICF-related form training activities received by the participants about the changes (if any). All analyses were performed using the SPSS software, version 25 (IBM Corporation, Armonk, NY, USA), with statistical significance set at *p* < 0.05.

## 3. Results

### 3.1. Changes in the Perceived Valences at Posttest

Table 2 presents the paired *t*-test results regarding the differences in the participants’ mean scores at pretest and posttest for the various ICF-related valence domains. Results show the average posttest scores were higher in all valence domains than the average pretest scores, and all of the differences were statistically significant (*p* < 0.01). In other words, the participants achieved significant positive changes in all valence domains after they had tried out the ICF-based practices for a year. 

### 3.2. Potential Personal Factors Influencing the Changes in the Participants’ Valences

Each personal factor (including gender, age, position, and years of experience in services for people with ID and service settings) was analyzed with one-way repeated-measures ANOVAs to examine if any of them has a significant impact on the difference between pretest and posttest. The level of involvement in the ICF-based practices and the number of training activities received were also assessed as separate covariates. Table 3 summarizes the results of one-way repeated-measures ANOVAs. Only the level of involvement in ICF-based practices had a significant effect, found in scores for the “Competence” domain, F (1, 87) = 5.651, *p* < 0.05, and “Intrapersonal” domain, F (1, 87) = 5.311, *p* < 0.05, as well as the “Total Score”, F (1, 87) = 4.000, *p* < 0.05. 

A follow-up bivariate Pearson Correlation analysis between the score differences and the level of involvement revealed that there are significant positive correlations with the level of involvement between the pre-post score differences in the “Competence” domain (*r* = 0.262, *p* < 0.05), the “Intrapersonal” domain (*r* = 0.242, *p* < 0.05) and the “Total Score” (*r* = 0.210, *p* < 0.05) of SSV-ICF (Table 4). Results show that the higher one’s level of involvement is in ICF-based practices, the more increments one will have of scores in the “Competence” domain, the “Intrapersonal” domain, and the “Total Score” of SSV-ICF after the one-year trial. 

## 4. Discussion

The findings of this study suggest the proven impact on staff members’ ICF-related valences of disability practitioners working under ICF-based practices. No matter what position was held (direct support worker or professional practitioner), the participants obtained positive changes in all domains of the valences. Results also show that the level of involvement by participants in the new ICF-based intervention had significant effects on score differences in the “Competence” domain, the “Intrapersonal” domain, and the “Total Score” of SSV-ICF after the one-year trial, but other personal factors and the number of training activities received did not have the same significant effect in any of the SSV-ICF domains.

After being involved in ICF-based practices for a year, the participating staff members’ knowledge of the ICF model and competence to apply the ICF framework in practices were significantly increased, no matter how many training activities they had received before. The increased “Intrapersonal” score indicates that participating staff members perceived that their jobs’ intrinsic attractiveness improved after the ICF-based practices were implemented. In previous research, the ICF framework has been proven to help clarify the roles of transdisciplinary team members [27,28]. The adoption of new ICF-based practices requires team members of different positions to work closely together in planning and evaluating interventions towards the shared goal of enhancing the “Activity” and “Participation” of service users. The documentation and meetings/conferences resulting from the new practices support team members of different positions and facilitate their connections, demonstrate their clinical reasoning, clarify their roles and responsibilities and improve communication within their teams [15,28,29]. The role clarity and value congruence arising from shared goals are important factors affecting team members’ job satisfaction [30,31]. The improved fit between their positions/professions and their roles under the new ICF-based practices also has been shown to support team members in feeling more positive about the changes in practices [32], and that information matches the findings of this study that the “Relational” domain (referring to individuals’ gains from the working relationship with team members and service users) and the “Meaning” domain (referring to individuals’ internal beliefs and values in favor of the adoption of new ICF-based practices) were also found to be significantly higher at posttest. Other than their enhanced competence, the staff members also developed stronger beliefs that the ICF-based practices had a positive influence on both service users and the organization.

The study results show that the staff members with different positions, lengths of experience, and service natures all benefitted from experience with ICF-based practices. In addition, the higher their involvement in the practice was, the greater enhancement they saw in the “Competence” domain, the “Intrapersonal” domain, and the “Total Score” of SSV-ICF they attained. This reveals that ICF-based practice itself contributes to the staff valence enhancement, but personal factors of staff do not. This highlights the merit of the ICF model in developing the human capital of disability services organizations. Through being involved in the new practice, staff members not only became more knowledgeable and competent with ICF practice at the personal level but also enhanced their effective working relationships with service users and colleagues from diverse positions at the interpersonal level. Furthermore, it helped staff members place more value on service users’ well-being and on the service enhancement of their own organization.

Disability services organizations in Hong Kong have encountered a relatively unfavorable situation in promoting the ICF model and related practices due to two main factors: Firstly, the lack of a top-down implementation approach by the government [33], secondly, perceived resistance from staff members towards changes in services [25]. From the perspective of behavior changes [34], the study results reflect that the pilot scheme implemented by the NGO may reduce staff members’ resistance by providing opportunities for staff members to try out ICF practices, and their reflective gains may, in return, motivate their acceptance of the change. A debriefing session for those participating staff members is recommended to the NGO in order to further facilitate staff insights and self-confidence. This would help consolidate a motivational enabler for the new ICF-based practice. 

For those NGOs planning to implement the ICF-based approach, it is recommended that after introducing the new approach, it is not only important to examine its impact on the staff perception regarding knowledge and competence but also particularly how it affects service users’ perception of the rehabilitation/inclusion process. Pre/post interviews or surveys with service users may also be beneficial to examine the impacts on service users.

## 5. Limitations

This study provides insights into the positive changes in staff valence after the adoption of the ICF model in an organization’s practices. A limitation should be noted that the findings reflect only the staff members’ cognitive reactions and perceptions towards the merits of ICF-based practices. Thus, we recommend that future studies explore the training needs of staff members and independently measure the changes in their skills and practical knowledge following the implementation of ICF-based practices. 

## 6. Conclusions

To the best of our knowledge, this study was the first attempt in the Asian context to address staff members’ perceptions of the merits of ICF-based practices. The hypothesis of this study proved that our findings demonstrate a positive impact of ICF-based practices on staff valences. From the ecological perspective, we believe that improving staff valences benefits service users and in so doing, also positively influences organizational culture. In its Rehabilitation Program Plan 2020 [32], the government of Hong Kong SAR claimed that it was exploring the adoption of the ICF framework in an effort to devise policies and interventions related to people with disabilities. At this beginning stage, a bottom-up strategy could benefit the development of ICF-based practices in Hong Kong. Furthermore, we hope that this study will facilitate additional related studies in the future and will encourage other disability organizations in Hong Kong to try adopting ICF-based practices.

## Figures and Tables

**Table 1:** Socio-demographic Characteristics of Participants (*N* = 91).

Variable	*n*	%
Gender		
Male	20	22.0
Female	71	78.0
Age, in years		
<21	0	0.0
21–30	10	11.0
31–40	23	25.3
41–50	36	39.6
51–60	20	22.0
>60	2	2.2
Position		
Social workers	36	39.6
Paramedical practitioners (therapists, psychologists, nurses, etc.)	31	34.1
Direct support staff (wardens, rehabilitation workers, vocational instructors, etc.)	24	26.4
Years of experience in services for users with intellectual disabilities		
10 or less	29	31.9
11–20	36	39.6
More than 20	26	28.6
Working in residential service setting		
Yes	65	71.4
No	26	28.6
Level of involvement in new ICF-based practices		
Involved in one component	59	64.8
Involved in two components	16	17.6
Involved in three components	16	17.6
Number of formal ICF-related training activities participated		
0	20	22.0
1	30	33.0
2	23	25.3
3	13	14.3
4	4	4.4
5	0	0.0
6	1	1.1

**Table 2:** Paired *t*-test results of various ICF-related valence domains.

ICF-Related Valence Domains	Pretest		Posttest		Difference		95% CI	*t/df*	*p* (2-tailed)	Effect size (Cohen’s *d*)
	M	SD	M	SD	M	SD				
Knowledge (*n* = 91)	15.69	4.46	17.60	3.68	1.91	3.90	[1.10, 2.72]	4.68/90	0.000	0.490
Competence (*n* = 91)	11.16	3.24	13.25	2.93	2.09	3.09	[1.45, 2.73]	6.45/90	0.000	0.677
Intrapersonal (*n* = 91)	26.48	5.77	28.10	5.17	1.62	5.55	[0.46, 2.77]	2.77/90	0.007	0.291
Relational (*n* = 89)	36.82	7.43	39.16	6.78	2.34	7.84	[0.69, 3.99]	2.81/88	0.006	0.298
Meaning (*n* = 91)	27.09	6.57	30.02	6.05	2.93	5.47	[1.80, 4.07]	5.12/90	0.000	0.537
Total (*n* = 89)	116.99	22.90	127.75	21.01	10.76	20.51	[6.44, 15.09]	4.95/88	0.000	0.525

**Table 3:** One-way Repeated measures ANOVAs results for the interaction effect of personal factors and covariates on scores of ICF-related valence domains.

ICF-Related Valence Domains	Personal Factors										Covariates			
	Gender		Age		Position		Years of Experience		Service Settings		Level of Involvement		Number of Formal Training	
	*F*(1, 87)	*p*	*F*(4, 84)	*p*	*F*(2, 86)	*p*	*F*(2, 86)	*p*	*F*(1, 87)	*p*	*F*(1, 87)	*p*	*F*(1, 87)	*p*
Knowledge	0.393	0.532	0.770	0.314	1.126	0.329	1.246	0.293	0.416	0.521	0.031	0.860	1.188	0.279
Competence	0.056	0.814	1.208	0.314	0.274	0.761	1.332	0.269	0.056	0.814	5.651	0.020 *	0.362	0.549
Intra-personal	0.622	0.432	1.169	0.331	0.589	0.557	0.816	0.446	1.638	0.204	5.311	0.024 *	1.989	0.162
Relation-al	0.109	0.742	1.423	0.233	1.123	0.330	1.794	0.174	1.885	0.173	3.659	0.059	0.752	0.388
Meaning	0.090	0.765	1.769	0.143	0.024	0.976	0.426	0.654	0.000	0.999	0.835	0.363	1.326	0.253
Total	0.027	0.870	1.647	0.170	0.449	0.640	1.335	0.269	0.610	0.437	4.000	0.049 *	0.822	0.367

* Significant at *p* < 0.05.

**Table 4:** Bivariate Pearson Correlation between the score differences in various ICF-related domains and the level of involvement.

Variables	1	2	3	4	5	6	7
Knowledge	---						
2. Competence	0.669 **	---					
3. Intrapersonal	0.509 **	0.445 **	---				
4. Relational	0.475 **	0.363 **	0.733 **	---			
5. Meaning	0.466 **	0.542 **	0.428 **	0.471 **	---		
6. Total score	0.740 **	0.687 **	0.834 **	0.856 **	0.738 **	---	
7. Level of involvement	0.023	0.262 *	0.242 *	0.201	0.348	0.210 *	---

** Significant at *p* < 0.001 (2-tailed), * Significant at *p* < 0.05 (2-tailed).

## Data Availability

The data presented in this study are available on request from the corresponding author.
